# Influence of Foot Type on the Clinical Outcome of Minimally Invasive Surgery for Metatarsalgia. A Prospective Pilot Study

**DOI:** 10.3389/fsurg.2021.748330

**Published:** 2021-09-21

**Authors:** Carmen Naranjo-Ruiz, Alfonso Martínez-Nova, María de los Ángeles Canel-Pérez, Miguel López-Vigil, Javier Ferrer-Torregrosa, Carlos Barrios

**Affiliations:** ^1^Doctorate School, Catholic University of Valencia “San Vicente Mártir”, Valencia, Spain; ^2^Podiatry Department, Catholic University of Valencia “San Vicente Mártir”, Valencia, Spain; ^3^Nursing Department, Podiatric Clinic of the University of Extremadura, Plasencia, Spain; ^4^Podocen, Madrid, Spain; ^5^Clinica Vigil, Gijón, Spain; ^6^Institute for Research on Musculoskeletal Disorders, Catholic University of Valencia “San Vicente Mártir”, Valencia, Spain

**Keywords:** foot, posture, metatarsalgia, osteotomy, metatarsal bones, minimally invasive surgical procedures

## Abstract

**Background and aims:** Surgical procedures for central metatarsalgia seek to harmonise the metatarsal parabola with osteotomies that can be performed by minimally invasive techniques. However, the possible relationship of the foot type and the mid-term postoperative outcome is poorly described. The objective of this prospective pilot study was therefore to determine whether the foot type (pronate, neutral, or supinate) conditions the postoperative mid-term functional outcome.

**Methods:** A series of 28 patients (6 men, 22 women) were treated for primary central metatarsalgia by means of minimally invasive distal metaphyseal osteotomy (DMMO).

**Results:** Their functional outcomes at 6 and 12 months were assessed by the self-reporting AOFAS scale. Pre-surgery, the patients' scores were 42.82 ± 15.60. Scores improved at 6 months to 86.50 ± 8.6 and to 92.93 ± 8.6 at 12 months (*p* < 0.001 in both cases). There were no differences either by sex or by foot type in these overall values, although there was only a slight limitation of interphalangeal mobility in the supinated feet (*p* = 0.03) at 6-month follow-up as compared to other foot types.

**Conclusion:** Hence, DMMO provides an optimal clinical and functional outcome for the surgical treatment of metatarsalgia, regardless of the patient's foot posture. The occurrence of adverse events was minimal and clinically irrelevant.

**Trial registration:** The study was authorised by the Research Ethics Committee of the Universidad Católica de Valencia San Vicente Mártir, with the registry UCV/2018-2019/019.

## Introduction

Metatarsalgia is a condition affecting the forefoot, characterised by pain under the central metatarsal heads that may also be associated with skin lesions (1). When the pain is related to alteration in the length of the metatarsals [mainly the second (2)] the term “dynamic metatarsalgia” is used (3–6). The prevalence of metatarsalgia is about 10% in the population, with female preponderance (7). An estimated 80% of the general population may experience symptoms of metatarsalgia at some point in their life. Biomechanical alterations are the main aetiological factor, accounting for 92 % of all aetiological factors (8).

There is disagreement among studies regarding the condition's ætiology. Some indicate that the presence of a longer metatarsal corresponds to excess loading, causing an increase in the plantar pressure under the affected metatarsal head (1, 3, 4, 9), and consequent metatarsal pain, while others find no such correspondence (8–10). With respect to the relative lengths of the metatarsals, (11) established a radiographic criterion for the surgical approach to forefoot pathology in which the length of the central metatarsals should be harmonised with that of the first metatarsal. In 62% of the group they studied with hallux valgus, metatarsals four and five presented hypoplasia, with the consequent exaggerated lengths of metatarsals two and three leading to metatarsalgia in this zone.

The presence of central metatarsalgia may be related to structural alterations of the foot, although there is no clear relationship of its prevalence with flat or cavus feet, or with other disorders such as intermetatarsal neuromas which have not been found to be more frequent in any specific posture or form of the foot (12). It may also be related to other biomechanical alterations, such as functional or anatomical equinus during the second rocker of the cycle and at the beginning of the propulsive phase (13). In attempt to unify all these etiological and biomechanical factors that cause metatarsalgia (excessive length or plantarflexion of one or more metatarsals, equinus foot, cavus foot, protrusion of a metatarsal head) and in order to address the treatment, whether conservative or surgical, this type of metatarsalgia is classified as primary metatarsalgia (1, 3). Other alterations, such as flat or pronated foot, have been related to the appearance of painful symptoms (14, 15). These alterations are also indicators of difficulties in performing tasks involving weight-bearing, so that a structural-functional evaluation must be taken into account in assessing what treatment to perform (16).

Despite the good outcomes provided by the commonly used conservative treatments (17–21), such as stretching exercises, shoe modifications, accommodative insoles or orthotics with arch support, these measures have low levels of recommendation (Level I) as options for treatment (1, 3, 4). When conservative treatment fails, surgical treatment is frequently implemented (22, 23), with the aim of restructuring the metatarsal parabola (24) by shortening the affected (and sometimes some of the adjacent) metatarsals (25). Various studies have proven the efficacy of minimally invasive distal metaphyseal osteotomy (DMMO). For example, (26, 27) achieved post-operative AOFAS scale scores of 86.5 and 88 with DMMO, as against 85.3 and 86, respectively, obtained with Weil's osteotomy. Indeed, AOFAS scores of up to 95.26 points have been achieved with DMMO (25). Similarly, studies that used another scale–the Manchester-Oxford Foot Questionnaire–have also reported acceptable (28) or good (29) improvements, with outcomes reaching a score of 31 (the lower the score, the better the outcome) in isolated metatarsal surgery (5).

However, to the best of the authors' knowledge, the relationship that the foot type may have to the post-operative outcome in the medium term remains unknown. Our hypothesis for this study is that clinical and functional outcomes after minimally invasive surgery for central metatarsalgia could be influenced by the foot morphology type (supinated, neutral or pronated) in accordance with the Foot Posture Index. Previous baropodometric research has found that the centre of head metatarsal pressures varies during walking in accordance with the spectrum of foot types (30). The larger area of lateral centre-of-pressure excursion was found in supinated feet, and the smaller in healthy people with pronated feet. It therefore seems relevant to analyse whether the variations of plantar kinematic related to foot morphology could have an impact on the outcomes after surgery for central metatarsalgia. This issue has never been addressed in the literature.

Thus, the objective of this study was to determine whether the patient's foot type (supinate, neutral or pronate) influenced the functional outcome at 6 and 12 months after DMMO for primary metatarsalgia in the second and the third metatarsals.

## Materials and Methods

### Study Design

A prospective study of 12 months' duration was conducted on a cohort of patients who required surgical treatment for central metatarsalgia after failed conservative therapies. The sample consisted of 28 participants (6 men and 22 women), with a mean age of 57.8 ± 9.9 years.

### Inclusion and Exclusion Criteria

Those included were male and female patients over 18 years in age who attended the centre where the study was carried out, this centre was the Podocen clinic, in Madrid; subjects recruitment was carried out in the time period April-June 2019, with a diagnosis of central metatarsalgia, which was localised in the second and third metatarsal heads. Patient recruitment occurred on a consecutive basis among the patients who had to have prior failed conservative treatment for at least a 1-year period. The subjects voluntarily agreed to participate, signing their informed consent to the corresponding intervention and study, and indicating that they would be available for follow-up at 6 and 12 months. The difference between the number of female vs. male subjects under study is a chance finding due to the consecutive recruitment of patients eligible for surgical treatment according to their admission to the centre where the study was conducted.

Patients who had undergone previous central metatarsal surgery, who had evident lower limb asymmetries, inflammatory arthropathies, paralysis of some lower limb muscle group, or hindfoot alterations (joint stiffness, osteoarthritis, posttraumatic deformity, e.g.,) that affected the overall position of the rear foot were excluded (more than 5° varus or valgus).

The study was authorised by the Research Ethics Committee of the Universidad Católica de Valencia San Vicente Mártir, with the registry UCV/2018-2019/019.

### Protocol

#### Measurement of Foot Posture

To classify each patient's foot type, the Foot Posture Index (FPI) was determined prior to surgery (31). The FPI is a validated method of quantifying foot posture, comprising 6 criteria based on observation of the forefoot and hindfoot with the patient standing. The hindfoot is evaluated by palpating the head of the talus, observing the curves above and below the malleoli, and the range of motion in inversion/eversion of the calcaneus. The forefoot is evaluated by observing the prominence of the talar-scaphoid joint, the congruence of the internal longitudinal arch, and the range of motion in abduction/adduction of the forefoot. Each item is scored from - 2 to 2, with the resulting total score being from - 12 to 12 (29, 30). The patients were classified into 3 groups: supinate (FPI - 12 to - 1, *n* = 13), neutral (FPI 0 to 5,13, *n* = 6), and pronate (FPI 6 to 12, *n* = 9).

#### Clinical Scale

The pertinent pre-operative tests were administered to each patient, and the AOFAS scale was completed including the minor metatarsophalangeal-interphalangeal joints subscale (32). The AOFAS scale assesses such subjective aspects as pain (characterised as severe, moderate, medium, or absent) and functional activity (severe limitations in daily activities, limitations in daily activities, limitations in recreational activities, or no limitations). These subjective values sum to a maximum of 60 points and, together with the examiner's objective analysis (40 points), constitute a measure of the patient's outcome (100 points) in terms of digital alignment, mobility, and stability. Scores of 90–100 are considered excellent, 72–89 good, 41–71 fair and below 40 poor. Scores of 91.0 or more would be values for individuals with no pathology, and so could be considered a threshold for the surgical treatment to be regarded as totally satisfactory (33).

#### Surgical Technique

**Figure 1:**
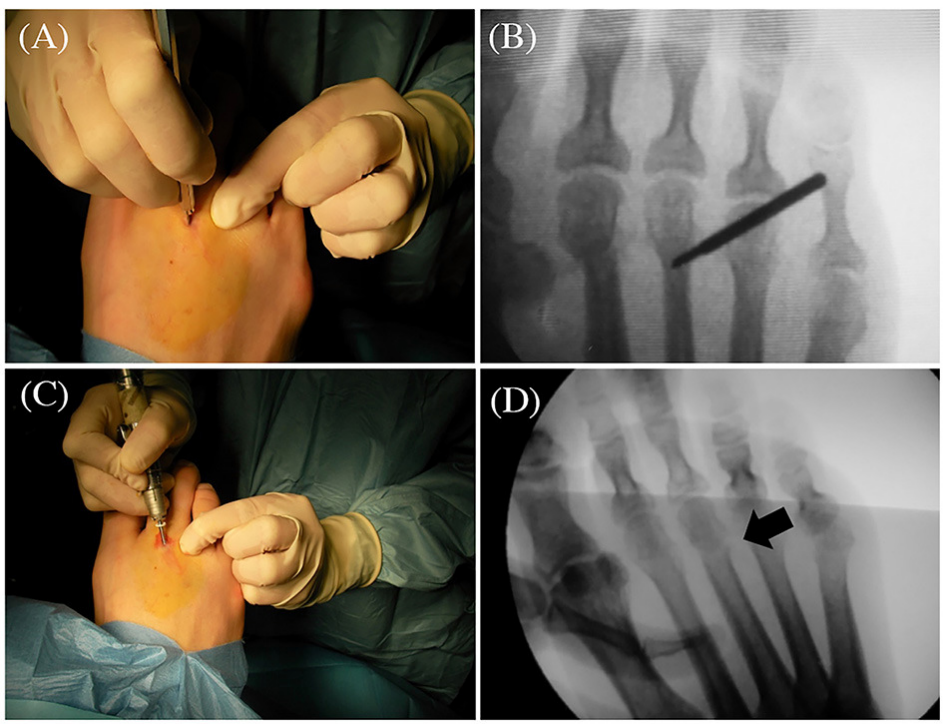
Details of the surgical technique. **(A)** Surgical incision of the 3rd metatarsal; **(B)** Position of the burr; **(C)** Performance of the osteotomy; **(D)** Fluoroscopic control of the metatarsal osteotomy.

For all the patients, the surgical procedure applied was DMMO of the 2nd and 3rd metatarsals. All patients were operated on an outpatient basis, the anaesthetic protocol consisted of an anaesthetic ankle block using 2% mepivacaine, as none of the patients were allergic to this drug. A 2-mm incision was made laterally and parallel to the extensor digitorum longus muscle with a Beaver #64 scalpel, at the level of the metatarsal head (Figure 1A). The incision was deepened at 45° angulation until the metatarsophalangeal capsule was reached, advancing the incision at the same angle to open the capsule and reach the cortex of the metatarsal neck. The cortex was marked at this point with the scalpel blade under fluoroscopic imaging to avoid unwanted movements of the burr when cutting (Figure 1B).

A micromotor with rpm control and a speed reduction and high-torque handpiece were used to minimise bone damage at the time of osteotomy. The burr used was an Isham Straight Flute Shannon 2.0 x 12.0 (mm) (Vilex Inc., McMinnville, Tennessee, USA). The angulation of the osteotomy was 45° with respect to the metatarsal's diaphyseal axis, with the burr direction intracapsular from distal dorsal to proximal plantar. The osteotomy was started on the lateral of the metatarsal neck with 45° angulation, ending the cut dorsally with the burr positioned perpendicular to the metatarsal axis and parallel to the metatarsal articular facet (Figures 1C,D).

The incision was closed with a single discontinuous suture of 4/0 monofilament nylon, and a bandage was applied using strips of Hypafix non-woven tape (BSN medical GmbH, Hamburg, Germany) to maintain fixation of the osteotomy. The patients were allowed to walk wearing a rigid sole, full support post-operative shoe (Darco International, Huntington, WV, USA). The first follow-up under fluoroscopy was performed at 72 h after surgery, and then at weekly visits to change the bandaging for 4–6 weeks until consolidation of the osteotomy was confirmed under the fluoroscopy. There are variations in the way of performing DMMO osteotomies. In this study, we used the intracapsular osteotomy, as in the López-Vigil study (25).

#### Follow-Up

Post-operative follow-up was carried out according to professional criteria. Patients did not follow any postoperative physical therapy program and they did not received any other additional treatment. The patients were asked to visit the centre where the procedure had been performed for AOFAS scale measurements at 6 months and at 12 months post-intervention. These periods represent normalisation of gait after the disappearance of residual œdema and any type of pain related to the intervention (25, 34).

### Statistical Analysis

With the sample comprising fewer than 30 subjects, the Shapiro-Wilk test was used to check for the normality of the data. For all the parameters, both pre-operative and post-operative, *p*-values < 0.05 were found, evidence that the data were not normally distributed. Thus, non-parametric statistical tests were used: (i) the Wilcoxon signed-rank test (related samples) for comparisons of the AOFAS scores at the three moments (prior to surgery, and 6 and 12 months post-surgery); and (ii) the Mann-Whitney U test for comparison of the AOFAS scores by sex (independent samples). The Kruskal-Wallis test was used for comparison of the AOFAS score with the overall classification of the groups of feet and in each of the dimensions of the AOFAS scale. The significance level was set at *p* < 0.05. All statistical tests were performed with SPSS vn 25 (SPSS INC, IBM, Chicago, IL).

## Results

### Sample Characteristics

Table 1 displays the anthropometric and clinical profile of the total sample, stratified by sex. As expected, males were heavier and taller than females (*p* < 0.05 and *p* < 0.01, respectively). However, there were no differences in BMI or foot laterality. The FPI assessment showed that the most frequent foot morphology type was the pronated (13 cases), followed by supinated (9 cases). Neutral feet accounted for only six cases. There were no statistically significant differences in the distribution of foot types by sex.

**Table 1:** Anthropometric and clinical characteristics of the sample.

	**Total sample** **(** ***n*** **= 28)**	**Males** **(** ***n*** **= 6)**	**Females** **(** ***n*** **= 22)**	**Mann-Whitney** **Z (** ***p*** **)**
Age (years)	57.8 ± 9.9	53.3 ± 7.2	59.0 ± 10.4	−1.234 (0.217)
Total mass (kg)	67.0 ± 11.9	79.4 ± 13.5	63.6 ± 9.1	−2.352 (0.019^*^)
Stature (m)	1.65 ± 0.09	1.78 ± 0.06	1.61 ± 0.06	−3.505 (0.000^**^)
BMI (kg/m^2^)	24.5 ± 3.4	24.9 ± 4.4	24.4 ± 3.2	−0.112 (0.911)
VAS (1-10)	7.7 ± 1.1	8.2 ± 0.7	7.5 ± 1.1	−1.459 (0.145)
Laterality (*n*/%)				
Right	16 (57.1)	4 (66.7)	12 (54.5)	Chi-squar
Left	12 (42.9)	2 (33.3)	10 (45.5)	*P* = 0.479
FPI (*n*/%)				
Pronated	13 (46.4)	4 (66.7)	9 (40.9)	Fisher's test
Neutral	6 (21.4)	0	6 (27.3)	*P* = 0.315
Supinated	9 (32.1)	2 (33.7)	7 (31.8)	

*BMI, Body mass index; VAS, Visual analogic scale for metatarsal pain; FPI, Foot postural index*.

* *p < 0.05;*

** *p < 0.01*.

### Postoperative Adverse Events

In the mid post-operative period, 10 patients developed edema at the surgical area that had disappeared at the last planned follow-up. There were also two cases with residual pain – one due to severe alterations in the associated hindfoot pathology (pronated foot), and the other due to excessive mechanical demands required by his work situation. No patient presented a delay in bone healing, non-union, floating toe, or stiffness of the metatarsophalangeal joint. At the end of follow-up, only one patient exhibit relapsed metatarsalgia for insufficient retraction and elevation of the metatarsal head. The limited number of complications does not seem to be related to the final result, since there is no significant statistical deviation in the final score of the AOFAS scale, as a whole or in any of its subscales (pain, alignment, function).

### AOFAS and VAS Scores for the Overall Sample and According to Sex

**Figure 2:**
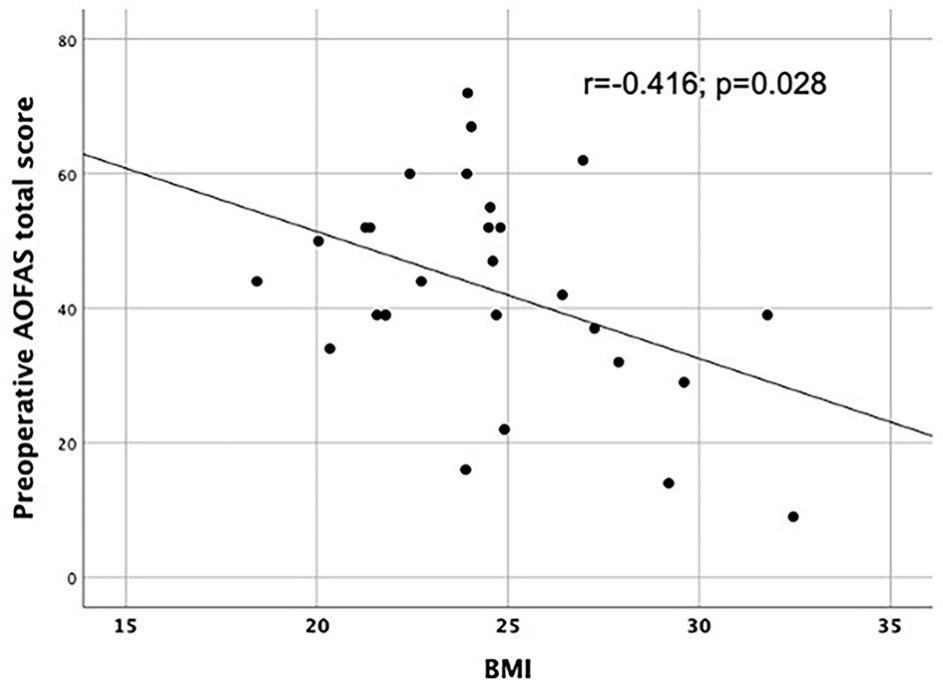
Correlation between AOFAS total scores and BMI in the whole sample.

The overall sample's pre-operative AOFAS score on the clinical scale was 42.8 ± 15.6. At 6 months it had increased significantly to 86.5 ± 8.6 (*p* < 0.001) and at 12 months to 92.9 ± 8.6 (*p* < 0.001). On the functional scale, the results showed no significant differences by sex. The pre-operative scores were 41.5 ± 14.6 in females and 47.5 ± 19.5 in males (*p* = 0.339). At 6 months, they were 85.9 ± 9.6 in females and 88.7 ± 3.5 in males (*p* = 0.715), and at 12 months, 92.4 ± 9.6 in females and 94.7 ± 2.9 in males (*p* = 0.752). AOFAS total scores before surgery corelated with BMI (*r* = - 0.416; *p* = 0.028); that is, higher BMI with corresponded lower AOFAS scores (Figure 2).

Concerning metatarsal pain assessed by the Visual analogic scale (VAS), the mean preoperative score for the whole sample was 7.7 ± 1.0, 0.7 ± 1.1 at 6-month follow-up and 0.3 ± 0.9 at 12-month follow-up. Except for the preoperative scores, males referred lower VAS scores than females at the two assessment stages of the follow-up, but without statistically significant differences (males vs. females preoperative scores: 8.2 ± 0.7 vs. 7.5 ± 1.1; 6-month scores: 0.6 ± 0.7 vs. 0.7 ± 1.1; 12-month scores: 0.0 ± 0.0 vs. 0.4 ± 1.0).

### AOFAS and VAS Scores by Foot-Type Group

Table 2 shows the preoperative AOFAS scores and those at 6 and 12-month follow-up, discriminating by foot morphology type. There were no significant differences in total AOFAS between the foot types at the preoperative assessment (*p* = 0.581), neither at 6-month (*p* = 0.352) nor 12-month follow-up (*p* = 0.979). Table 2 presents also the results of the 3 AOFAS subscales (pain, function and alignment) scored by foot type. There were no differences among the 3 foot types at any of the check-ups. However, when scores were analysed by comparing pairs, supinated feet showed fewer functional limitations than to neutral feet (Mann-Whitney test; Z:-2.152 *p* = 0.031). Supinated feet showed also a trend towards, less preoperative pain (higher AOFAS pain scores), but without statistical significance. Supinated feet showed more misalignment (lower AOFAS alignment scores) than pronated feet (Mann-Whitney test; Z: - 1.943; *p* = 0.049). In the preoperative evaluation, AOFAS scores for function correlated positively with AOFAS alignment scores (*r* = - 0.525; *p* = 0.004).

**Table 2:** AOFAS scores and metatarsal pain assessed by the VAS according to foot type.

	**Pronated** **(** ***n*** **= 13)**	**Neutral** **(** ***n*** **= 6)**	**Supinated** **(** ***n*** **= 9)**	**Kruskal-Wallis** **H (** ***p*** **)**
**AOFAS Total**				
Preop.	40.8 ± 17.2	39.3 ± 17.1	48.0 ± 12.3	1.085 (0.581)
6-month FU	89.3 ± 6.1	85.5 ± 7.8	83.1 ± 11.1	2.089 (0.352)
12-mont FU	94.2 ± 5.3	92.6 ± 9.3	91.2 ± 12.2	0.043 (0.979)
**AOFAS Pain**				
Preop.	10.7 ± 10.4	16.7 ± 8.2	20.0 ± 8.7	(5.184) 0.075
6-month FU	36.1 ± 5.1	35.0 ± 5.5	33.3 ± 7.1	(0.948) 0.622
12-mont FU	38.5 ± 3.8	36.6 ± 5.2	37.8 ± 6.7	(1.034) 0.596
**AOFAS Function**				
Preop.	22.5 ± 8.1	20.0 ± 7.4	26.6 ± 7.1^*^	3.057 (0.271)
6-month FU	39.6 ± 3.9	39.0 ± 1.5	38.7 ± 4.7	0.622 (0.733)
12-mont FU	41.8 ± 3.5	43.3 ± 2.5	40.8 ± 4.1	1.728 (0.422)
**AOFAS Alignment**				
Preop.	7.1 ± 6.5	2.7 ± 4.2	2.6 ± 5.4^**^	(3.889) 0.143
6-month FU	13.9 ± 2.6	11.5 ± 3.8	11.1 ± 3.7	(4.259) 0.119
12-mont FU	13.9 ± 2.6	12.7 ± 3.6	12.7 ± 3.5	(1.154) 0.562
**VAS**				
Preop.	8.1 ± 1.1	7.8 ± 0.9	7.1 ± 0.9^***^	5.833 (0.054)
6-month FU	0.6 ± 0.9	0.4± 0.5	0.9 ± 1.5	0.322 (0.851)
12-mont FU	0.2 ± 0.7	0.2 ± 0.4	0.5 ± 1.4	0.830 (0.660)

*FU, follow-up*.

* *As compared to neutral feet (Mann-Whitney test; Z:-2.152; p = 0.031)*.

** *As compared to pronated feet (Mann-Whitney test; Z:-1.943; p = 0.049)*.

*** *As compared to pronated feet (Mann-Whitney test; Z:-2.103; p = 0.035)*.

**Figure 3:**
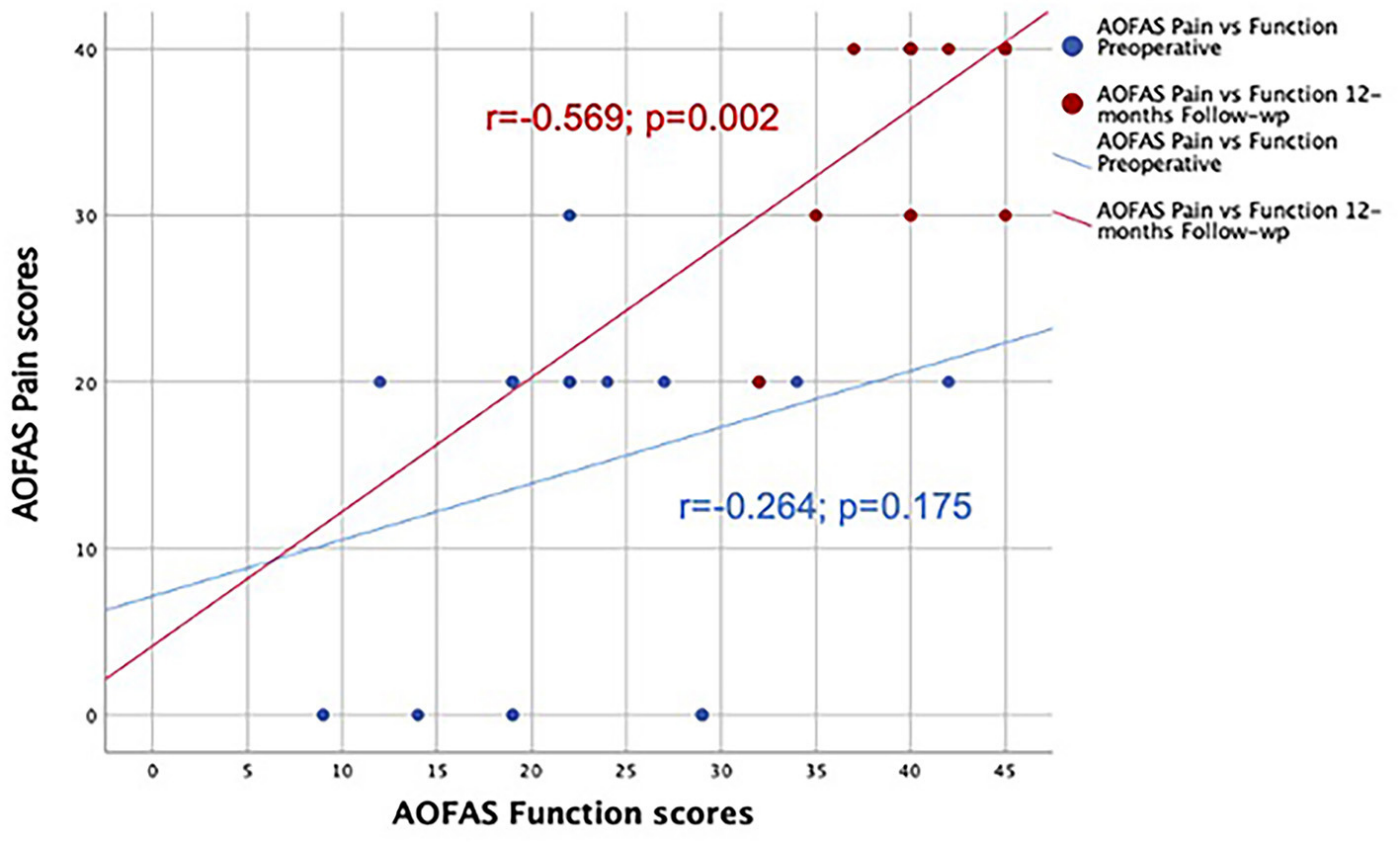
Positive correlation between AOFAS Pain and function scores at 12-month follow-up, but not at the preoperative assessment.

As to VAS scores for the whole sample, the improvement induced by the surgical treatment was clearly evident for all foot types. Similar to AOFAS pain scores, supinated feet showed lower VAS scores than pronated feet (Mann-Whitney test; Z:- 2.103; *p* = 0.035). There was a good correlation between AOSFAS pain subscale and WAS scores before surgery (*r* = - 0.674; *p* = 0.000) and 12 months after surgery (*r* = - 0.888; *p* = 0.000). AOFAS scores for pain correlated with function scores at the 12-month follow-up, but not at the preoperative assessment (Figure 3).

The detailed results of the different dimensions of the AOFAS function subscale are shown in Table 3. In the preoperative evaluation, neutral feet exhibited almost complete Metatarsal-phalangeal joint MTP joint motion. Differences with supinated feet were statistically significant (Mann-Whitney test; Z:4.667; *p* = 0.031). The restoration of the IP joint motion was not complete in supinated feet. In fact, at 6 months the supinate feet presented less interphalangeal mobility (3.3 points) than the neutral (5 points) or pronate (5 points) feet (Table 3, *p* = 0.033). There were no differences between foot types for the remaining parameters of the scale (*p* > 0.05 in all cases, Table 3).

**Table 3:** Dimensions of the AOFAS function subscale according to the foot type.

**AOFAS Function Subscale**		**Pronated** **(** ***n*** **= 13)** **Mean**	**Neutral** **(** ***n*** **= 6)** **Mean**	**Supinated** **(** ***n*** **= 9)** **Mean**	**Kruskal-Wallis** **H (** ***p*** **)**
Activity Limitations (10 points)	Pre-	5.1 ± 1.5	5.5 ± 1.6	5.7 ± 1.6	(0.649) 0.726
	6 months	8.8 ± 1.5	9.0 ± 1.5	8.7 ± 1.6	(0.186) 0.911
	12 months	9.5 ± 1.1	10.0 ± 0.0	9.7 ± 1.0	0.982) 0.612
Footwear requirements (10 points)	Pre-	4.6 ± 1.4	4.2 ± 2.0	3.8 ± 2.2	(0.918) 0.632
	6 months	6.1 ± 2.2	6.7 ± 2.6	6.1 ± 2.2	0.275) 0.872
	12 months	8.1 ± 2.5	7.5 ± 2.7	7.8 ± 2.6	(0.228) 0.892
MTP joint motion (10 points)	Pre-	5.8 ± 2.8	8.3 ± 2.6^*^	5.6 ± 1.7	(5.218) 0.074
	6 months	9.2 ± 1.9	10.0 ± 0.0	10.0 ± 0.0	(2.396) 0.302
	12 months	9.2 ± 1.9	10.0 ± 0.0	10.0 ± 0.0	(2.396) 0.302
IP joint motion (5 points)	Pre-	1.9 ± 2.5	4.2 ± 2.0	1.7 ± 2.5	(4.086) 0.130
	6 months	5.0 ± 0.0	5.0 ± 0.0	3.3 ± 2.5	(6.840) 0.033^**^
	12 months	5.0 ± 0.0	5.0 ± 0.0	3.9 ± 2.2	(4.385) 0.112
MTP-IP Stability (5 points)	Pre-	5.0 ± 0.0	4.2 ± 2.0	3.9 ± 2.2	(2.920) 0.232
	6 months	5.0 ± 0.0	5.0 ± 0.0	5.0 ± 0.0	(0.000) 1.00
	12 months	5.0 ± 0.0	5.0 ± 0.0	5.0 ± 0.0	(0.000) 1.00
MTP-IP related callus (5 points)	Pre-	0.4 ± 1.4	1.7 ± 2.6	0.0 ± 0.0	(4.255) 0.119
	6 months	4.6 ± 1.4	5.0 ± 0.0	4.4 ± 1.7	(0.657) 0.720
	12 months	4.6 ± 1.4	5.0 ± 0.0	4.4 ± 1.7	(0.657) 0.720

*MTP, metatarsal-phalangeal joint; IP, interphalangeal joint*.

* *as compared to supinated feet (Mann-Whitney test; Z:4.667; p = 0.031)*.

** *statistically significant difference among the 3 foot types*.

## Discussion

That we were unable to detect differences between the 3 groups of feet despite the general improvement observed in the overall sample may be due to the great specificity of the AOFAS scale for minor interdigital and metatarsophalangeal joints. In evaluating a relatively small zone, it is possible either that the foot type has no influence on the surgical outcome or that this scale is unable to detect it.

The improvement in the dimensions of pain, functionality, and alignment were practically homogeneous across the 3 foot types. There is only poorer mobility of the interphalangeal toe joint in the supinate feet. This may be due to the great tendinous retraction of the supinate feet, which probably also present a morphology close to pes cavus. In this type of foot, the tendon retraction causes claw toes during the propulsive phase of gait, maintaining plantarflexion of the metatarsal head and the appearance of metatarsalgia (3, 13). This finding should make us focus future attention on improving this result. Subsequent investigations could perhaps add minimally invasive tenotomies that relax the dorsal tendinous retraction, even though they do not negatively impact the overall outcome, the aim being to counter the reduced mobility of this joint.

It is clear from the literature that DMMO, with follow-up periods similar to those applied in this present study, yields excellent results in patients with primary metatarsalgia. Indeed, this osteotomy seems to achieve outcomes comparable to those obtained with Weil's osteotomy (26, 27), while having the advantage of involving less limitation of the MTP joint's motion, possibly due to a reduction of soft tissue damage and of interference with the blood supply to the metatarsal head (35–37).

Second and third metatarsal DMMO has thus shown adequate efficacy to guarantee clinical and functional improvement in the patients of our cohort. The outcomes at 6 months were good (86.5 points), and at 12 months (92.9 points) were within the excellent range, with scores comparable to those of non-pathological feet (33). Our study's values were similar to those reported by López-Vigil (24) who, with similar minimally invasive techniques, were able to improve the clinical status from 50.3 points pre-operatively to 95.2 points at an average of 18 months after surgery. Both the present study and that by López-Vigil (25) reached higher post-operative scores than those for the DMMO technique reported by (27) with 88 points at 6 months, (24) with 84.1 at 58.7 months on average, and (26) with 86.5 points at 14 months after surgery.

With respect to the outcomes in the different dimensions of the AOFAS scale, in our cohort, metatarsophalangeal motion functionality presented post-operative scores at 6 and 12 months of 10.00 in the supinate and normal foot subgroups and 9.23 in the pronate foot subgroup. This indicates recovery of complete mobility, without the appearance of complications such as joint stiffness or floating toe. These findings are consistent with those of (24) and (29) who also reported the absence of persistent joint stiffness in their cohorts. Our results are significant improvements in metatarsophalangeal joint stiffness relative to those reported by (24, 26, 27, 38), with a variability of between 4 and 34% in their DMMO cohorts.

In our cohort, there were no differences by foot type in the recovery of metatarsophalangeal mobility after DMMO. This may be related to the choice of the point at which to perform the osteotomy, as it was done intracapsularly. This does not damage the plantar plate (25), and the reduced need for recovery of the soft tissues surrounding the joint could be related to the absence of post-operative stiffness at 6 and 12 months.

Neither did the foot type appear to have any influence on the post-operative recovery of the central ray alignment dimension of the AOFAS scale. The scores went from starting values of 7.08 in the pronate foot, 2.67 in the neutral foot, and 2.56 in the supinate foot to values at the end of the study (12 months) of 13.92, 12.67 and 12.67, respectively. Our results for neutral and supinate feet were slightly lower than the mean score of 13.7 for non-pathological feet reported by (32). In the supinate feet, this could be due to the greater tendinous retraction with the consequent deviation of the middle toes, but a decrease in the values of just 1 point can also be considered a deviation reflecting the mean age of the patients.

In summary, the improvement in all the dimensions of the AOFAS scale, comparing the values to those obtained in feet without pathology, confirms that minimally invasive surgery improves pain, completely recovers mobility and stability of the metatarsophalangeal joints and allows patients to recover the use of conventional footwear in the medium term. This study has obtained better results on the AOFAS scale than similar studies of DMMO and Weil osteotomies, which confirms the minimally invasive osteotomy performed intracapsularly as a more recommendable option for patients due to the minimal number of complications.

This study has shown that there is no difference between foot type and the short- and mid-term outcome of surgical treatment of metatarsalgia with minimally invasive osteotomies, so that in clinical practice there is no need to adjust treatment in patients with pronated or neutral feet as the treatment works fully in all dimensions of the AOFAS scale. These findings are not influenced by the sex of the study subjects, as no significant differences were found between the two genders. However, the lesser improvement of the interphalangeal joint mobility dimension indicates the need to add another treatment to the affected toes to achieve full interphalangeal joint mobility of the toes.

### Study Limitations

A limitation of the study could be the limited number of patients recruited in the cohort. Nevertheless, this is a pilot study; therefore, the number of participants can be considered sufficient to obtain promising results. Pilot studies do not require usually a determined calculation of the sample size. Another limitation is that the follow-up data is limited to 12 months after surgery, although this fact prompts us to continue the follow-up of these patients in order to assess the outcomes over a longer term. While there is a degree of controversy on the part of some authors over the use of the AOFAS scale (39, 40), it is still widely used as a clinical tool among foot surgeons (41, 42) and, in the present study, was disaggregated into each of its sections–pain, functionality, and alignment–in order to try to find the possible relationship between the different foot types according to the IPF and the improvements in outcome obtained after the intervention.

## Conclusions

Independent of the foot type, DMMO performed at the 2nd and 3rd metatarsal provides satisfactory functional results, as shown by the improvement of the scores in the different dimensions of the AOFAS scale evaluated post-operatively at 6 and 12 months. At the end of follow-up, the AOFAS results were superior to those reported in the medical literature concerning metatarsal osteotomies. The current AOFAS results can be considered similar to those obtained in non-pathological feet. In addition, there was no distinction in the clinical and functional final scores according to the foot type of the patients in the cohort. The only difference was found in supinated feet, which showed a delay in the post-surgical recovery of the interphalangeal mobility at 6-month follow-up as compared to other foot types. Further research enlarging the sample size is required to confirm the promising results of DMMO technique for surgical correction of primary central metatarsalgia based on the few complications and the satisfactory clinical and functional outcomes found in the current series.

## Data Availability Statement

The datasets used and/or analyzed during the current study are available from the corresponding author on reasonable request.

## Ethics Statement

The study was authorised by the Research Ethics Committee of the Universidad Católica de Valencia San Vicente Mártir, with the registry UCV/2018-2019/019. This clinical practice observational study was performed in accordance with the ethical standards of the 1964 Declaration of Helsinki as revised in 2000 and those of Good Clinical Practice. All patients received a thorough explanation of this study and written informed consent to participate was obtained from the participants.

## Author Contributions

CN-R performed the surgeries, analyzed, and interpreted the data. JF-T contributed to the study concept and design, and participated in the figure designs. CN-R, MC-P, and ML-V carried out the data collection and analysis. AM-N contributed to the study concept and design, and the interpretation of data. CB participate in the study design, the statistical analysis, and was a major contributor in writing the manuscript. All authors read, discuss, and approved the final manuscript. The authors declare that this article represents honest work.

## Conflict of Interest

The authors declare that the research was conducted in the absence of any commercial or financial relationships that could be construed as a potential conflict of interest.

## Publisher's Note

All claims expressed in this article are solely those of the authors and do not necessarily represent those of their affiliated organizations, or those of the publisher, the editors and the reviewers. Any product that may be evaluated in this article, or claim that may be made by its manufacturer, is not guaranteed or endorsed by the publisher.
